# Endoplasmic reticulum stress activates telomerase

**DOI:** 10.1111/acel.12161

**Published:** 2013-10-22

**Authors:** Junzhi Zhou, Beibei Mao, Qi Zhou, Deqiang Ding, Miao Wang, Peng Guo, Yuhao Gao, Jerry W Shay, Zengqiang Yuan, Yu-Sheng Cong

**Affiliations:** 1 Institute of Aging Research, Hangzhou Normal University School of MedicineHangzhou, 310036, China; 2 Key Laboratory for Cell Proliferation and Regulation Biology of Ministry of Education, Beijing Normal UniversityBeijing, 100875, China; 3 Institute of Biophysics, Chinese Academy of SciencesBeijing, 100101, China; 4 Department of Cell Biology, UT Southwestern Medical CenterDallas, TX, 75390-9039, USA

**Keywords:** apoptosis, ER stress, telomerase, hTERT

## Abstract

Telomerase contributes to cell proliferation and survival through both telomere-dependent and telomere-independent mechanisms. In this report, we discovered that endoplasmic reticulum (ER) stress transiently activates the catalytic components of telomerase (TERT) expression in human cancer cell lines and murine primary neural cells. Importantly, we show that depletion of hTERT sensitizes cells to undergo apoptosis under ER stress, whereas increased hTERT expression reduces ER stress-induced cell death independent of catalytically active enzyme or DNA damage signaling. Our findings establish a functional link between ER stress and telomerase, both of which have important implications in the pathologies associated with aging and cancer.

Human telomerase is a ribonucleoprotein enzyme complex that is minimally composed of a RNA component (hTR or hTERC) and the telomerase reverse transcriptase (hTERT; Blackburn, 2000). Telomerase has fundamental roles in aging and in cancer (Artandi, 2006). However, the pathways and molecular mechanisms regulating telomerase remain poorly understood. Importantly, telomerase appears to have telomere-independent functions in a number of fundamental cellular processes (Cong & Shay, 2008). The endoplasmic reticulum (ER) is a cytosolic membrane network connected to the nucleus, mitochondria, and the plasma membrane (Tabas & Ron, 2011). The ability to respond to perturbations in ER function (ER stress) is a fundamental important property of cells (Wang & Kaufman, 2012).

We sought to investigate the expression and function of telomerase in ER stress responses. In HeLa and MCF7 cells, treatment with thapsigargin (Tg) or ionomycin (In) induced ER stress responses characterized by increased specific expression of Bip, IRE1α, CHOP, and *XBP-1* (Fig. 1A). Interestingly, we found that hTERT protein and *hTERT* mRNA expression was up-regulated 1 h after the Tg or In treatment, which then decreased 8 h post-treatment (Figs 1A,B and S1A). No changes were observed in the levels of hTR, (data not shown). Additionally, the expression of hTERT was increased in MCF7 cells under different ER stress conditions (Fig. S1B). As another proof that hTERT protein levels are increased under ER stress, we showed that the telomerase enzymatic activity *in vitro* was also increased (Figs 1C and S1C). Importantly, treatment with Tg dramatically increased the *mTERT* mRNA expression in primary mouse CGN and NPC cells (Figs 1D and S2). Control treatment with DMSO in HeLa, MCF7, and CGN cells has no effect on either ER stress induction or TERT up-regulation (Fig. S3). These results suggest that up-regulation of TERT under ER stress may represent a *bona fide* cellular process involved in the ER stress response.

**Figure 1:**
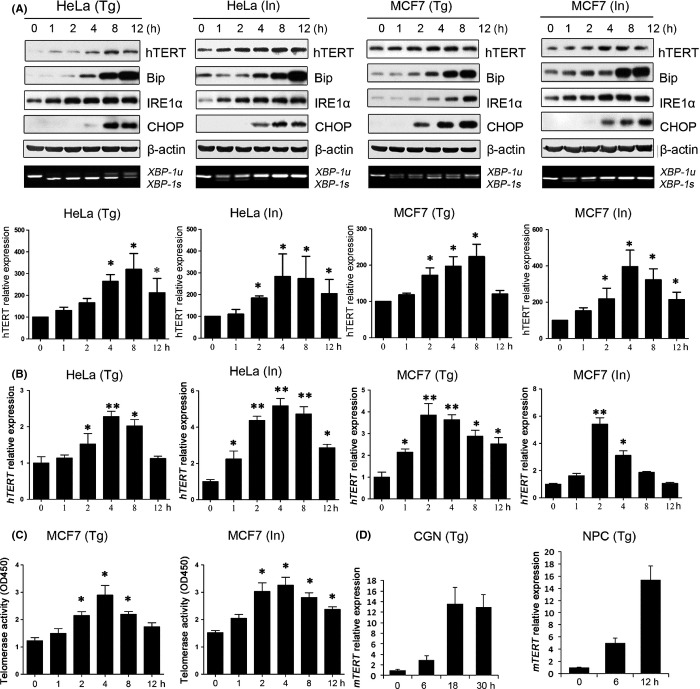
Up-regulation of TERT expression under ER stress. (A) MCF7 and HeLa cells were treated with 10 μm ionomycin (In) or 2 μm thapsigargin (Tg). At the indicated time points, cells were harvested for Western blot analysis of hTERT, Bip, IRE1α, and CHOP protein expression, respectively, and for RT–PCR analysis of *XBP-1u* and *XBP-1s* mRNA expression. β-actin was used as internal control. The experiment was repeated three times and a representative result is shown in upper panel. Level of hTERT expression quantified densitometrically from three independent experiments is shown in lower panel. (B) ER stress increased *hTERT* mRNA expression. MCF7 and HeLa cells were treated with 10 μm In or 2 μm Tg. At the indicated time points, cells were harvested for real-time PCR analysis of h*TERT* mRNA expression. *GAPDH* was used for normalization. (C) MCF7 cells were treated with 10 μm In or 2 μm Tg. At the indicated time points, cell lysates were prepared for assays of the telomerase activity using TRAPEZE® Telomerase Detection Kit. (D) Primary mouse CGN and NPC cells were treated at indicted times. Cells were then harvested for real-time PCR analysis of *mTERT* mRNA expression. *GAPDH* was used for normalization. Data are presented as the mean ± SD from three independent experiments (*P < 0.05,**P < 0.001). Detailed experimental procedures are described in Data S1.

We then investigated potential roles of TERT against ER stress-induced cell death. As shown in Fig. 2, treatment with Tg induced apoptotic cell death in MCF7 cells. However, the depletion of hTERT by four hTERT-specific On-Target plus siRNA oligos (Dharmacon) potentiated cell death of MCF7 cells under ER stress (Fig. 2A). The depletion of hTERT expression also increased Tg-induced cytotoxicity in MCF7 cells (Fig. 2B). Conversely, in U2OS cells that lack telomerase expression, infection of lenti-hTERT or lenti-K626A, a catalytically inactive mutant hTERT K626A (Weinrich *et al*., 1997), significantly inhibited cell death (Fig. 2C) and reduced cytotoxicity under Tg-induced ER stress (Fig. 2D). Together, these results indicated that increased expression of hTERT suppresses the ER stress-induced cell death independently of catalytic active telomerase enzyme.

**Figure 2:**
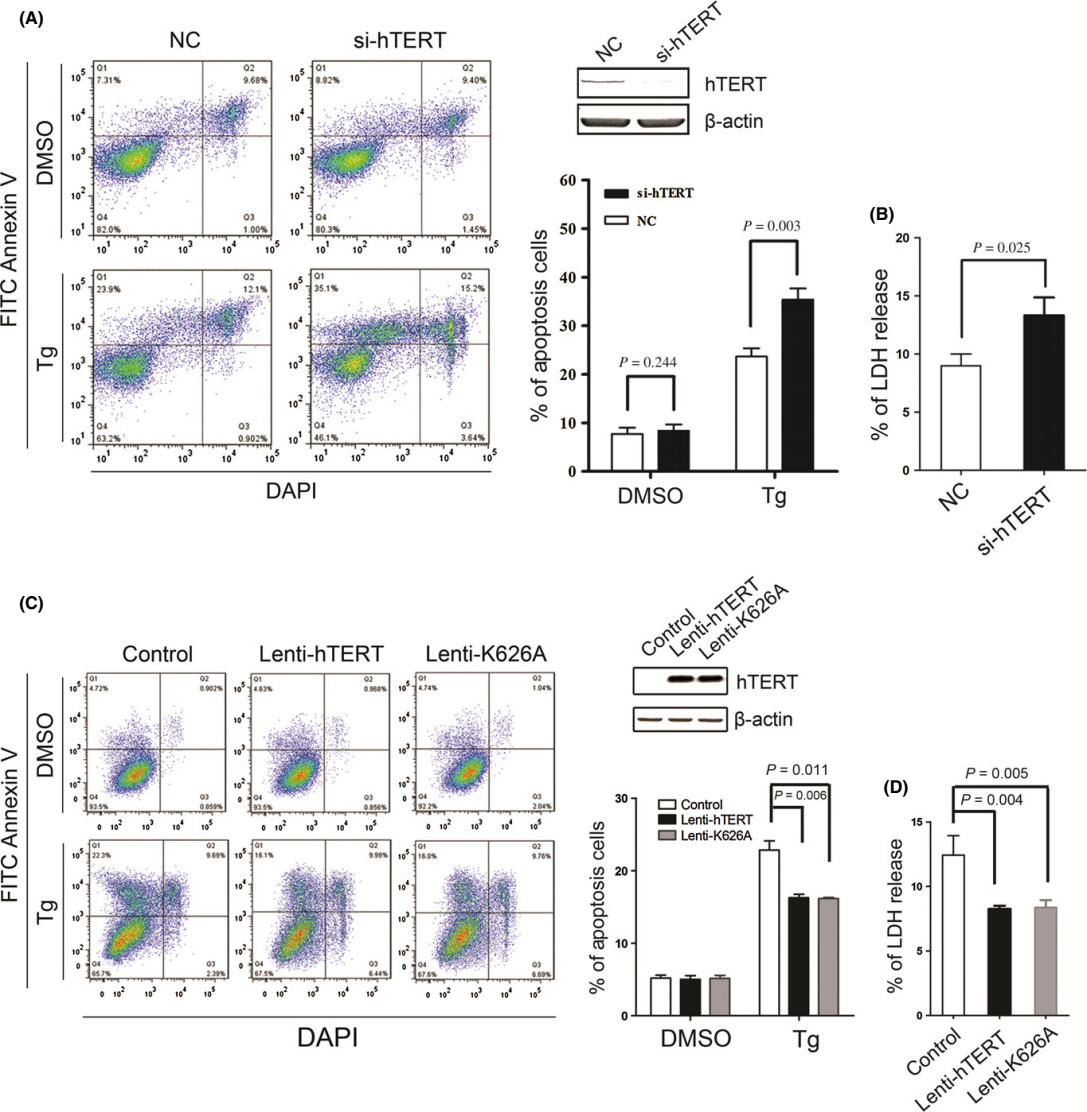
hTERT promotes cell survival under ER stress. (A) MCF7 cells were transfected by the control siRNA (NC) or by hTERT-specific siRNA (si-TERT). Forty-eight hour after transfection, cells were treated with 2 μm thapsigargin for 60 h, followed by bivariate flow cytometric analysis for the detection of apoptotic cells (AnnexinV+/DAPI−). Representative fluorescence-activated cell sorting pictograms and mean data ± SD (*n* = 3) are shown. The depletion of hTERT expression was confirmed by immunoblotting. (B) MCF7 cells were transfected by the control siRNA (NC) or by hTERT-specific siRNA (si-TERT) and treated as described in (A). The medium lactate dehydrogenase (LDH) activity was measured using the LDH Cytotoxicity Assay Kit. (C) U2OS cells were infected with lenti-hTERT, lenti-K626A or control virus. 48 h after infection, cell were treated with 2 μm Tg for 48 h, apoptotic cells were detected as described in (A). Representative fluorescence-activated cell sorting pictograms and mean data ± SD (*n* = 3) are shown. (D) U2OS cells were infected and treated as described in (A). The medium lactate dehydrogenase (LDH) activity was measured using the LDH Cytotoxicity Assay Kit. Detailed experimental procedures are described in Data S1.

We next tested if ER stress induced DNA damage response and subsequently activated telomerase. Our results indicated that ER stress under our assay conditions did not result in DNA damage responses (Fig. S4). Given that hTERT was up-regulated as early as 1 h after ER stress induction and decreased at 8 h postinduction, we therefore conclude that the anti-apoptosis effect of increased hTERT expression under ER stress may be independent of telomere maintenance.

hTERT was up-regulated immediately upon ER stress induction, suggesting transcriptional regulation. It is known that the ER stress pathway triggers activation of the transcription factor NF-κB (Hung *et al*., 2004) and that the transcription factor NF-κB has been implicated with the regulation of hTERT transcription (Sinha-Datta *et al*., 2004). We then investigated whether NF-κB was involved in up-regulation of hTERT expression under ER stress. We observed that ER stress increased nuclear translocation of the NF-κB p65 transcription factor, which correlated with a concomitant decrease in the IκBα levels and an increase in the NF-κB reporter activity (Fig. S5). However, the transfection of the IκBα super-repressor (IκBα-SR), an efficient NF-κB inhibitor (Reuther *et al*., 1998), inhibited the ER stress-induced hTERT up-regulation (Fig. S6A). Similarly, ER stress-induced hTERT up-regulation was compromised in the presence of the specific NF-κB inhibitor PDTC (Fig. S6B). These results indicate that the NF-κB pathway may be required for up-regulation of hTERT under ER stress.

TERT is expressed at high levels during the process of neuronal differentiation and then decreases sharply during the period when programmed cell death occurs (Fu *et al*., 2000). Importantly, suppression of TERT expression promotes apoptosis of neurons, whereas overexpression of TERT suppresses apoptosis (Fu *et al*., 2000). Additionally, fully differentiated neurons and astrocytes do not express telomerase activity, but the expression of TERT increased in response to oxidative stress, amyloid β-peptide-induced damage and ischemia-induced neurotoxicity (Saretzki, 2009). These observations suggest that deregulation of TERT expression due to genetic or epigenetic alterations in the adult brain may contribute to the vulnerability in age-related neurodegenerative disorders. In contrast, cancer cells frequently experience ER stress as results of hypoxia and nutrition deprivation conditions in the tumor microenvironment. Thus, high levels of hTERT expression associated with cancer may contribute to the resistance of cancer cells to regulation by the tumor microenvironment or in the responses to therapy. Our current findings establish a functional link between ER stress and telomerase, both of which have important implications in the pathologies associated with aging and cancer. Thus, understanding the relationship between ER stress response and telomerase induction may provide molecular insights into the mechanisms associated with ER stress-related disease.

## Supporting Information

**Fig. S1** ER stress up-regulates the expression of hTERT in cancer cells.

**Fig. S2** ER stress induction in mouse primary cells.

**Fig. S3** Effects of the control treatment with DMSO on ER stress induction and TERT up-regulation.

**Fig. S4** Absence of DNA damage response in cells under ER stress.

**Fig. S5** ER stress enhances the NF-κB activity.

**Fig. S6** The NF-κB is required for increased hTERT expression under ER stress.

**Data S1** Experimental procedures.
