# Supplementary figures and images for: Endoplasmic reticulum stress activates telomerase
Source: Aging Cell. 2013 Oct 22;13(1):197–200. doi: 10.1111/acel.12161 (PMC4326870; doi:10.1111/acel.12161)

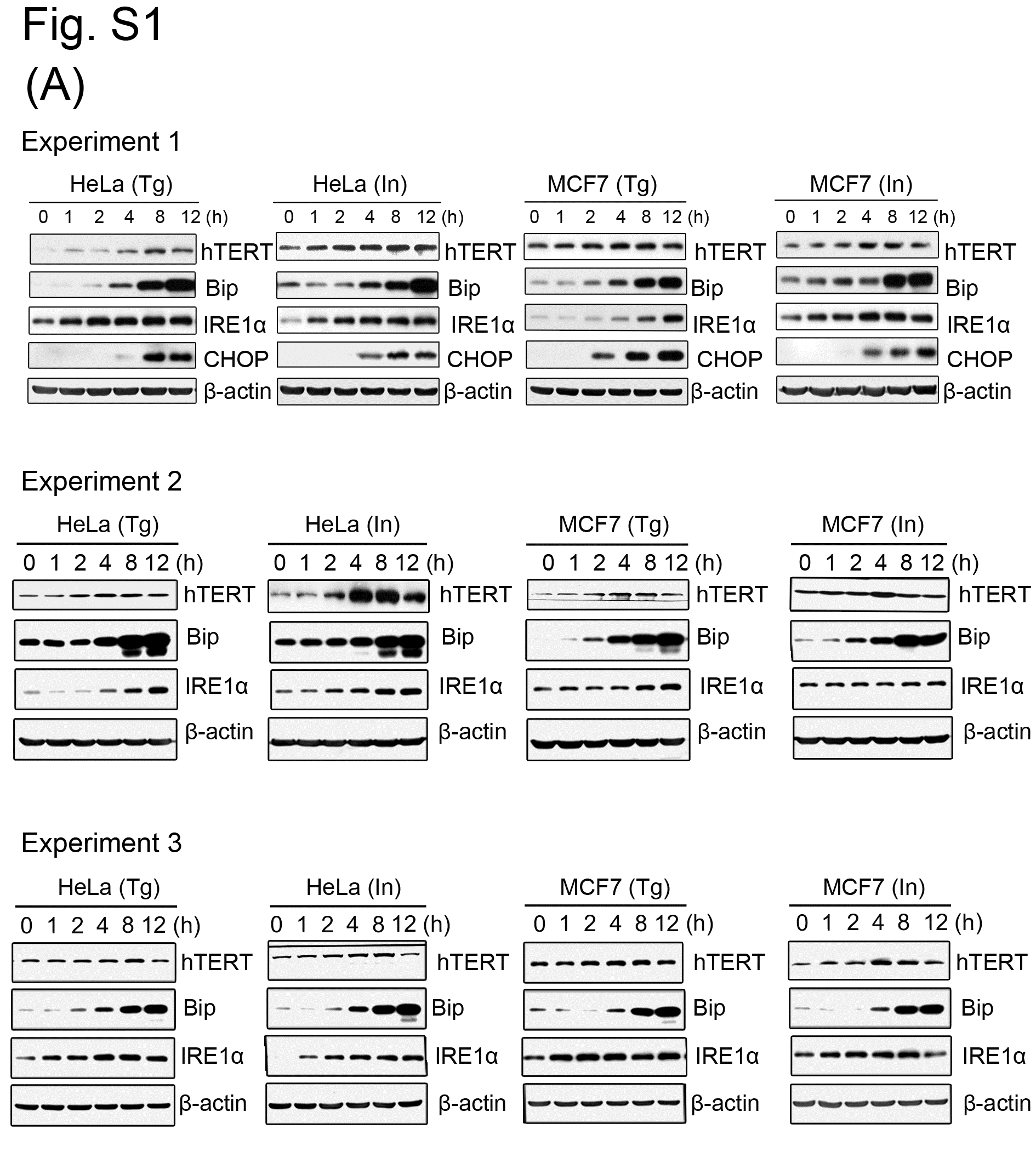

Supplement: Supplementary file 1 — Fig. S1 ER stress up-regulates the expression of hTERT in cancer cells. [file acel0013-0197-sd1.tif]

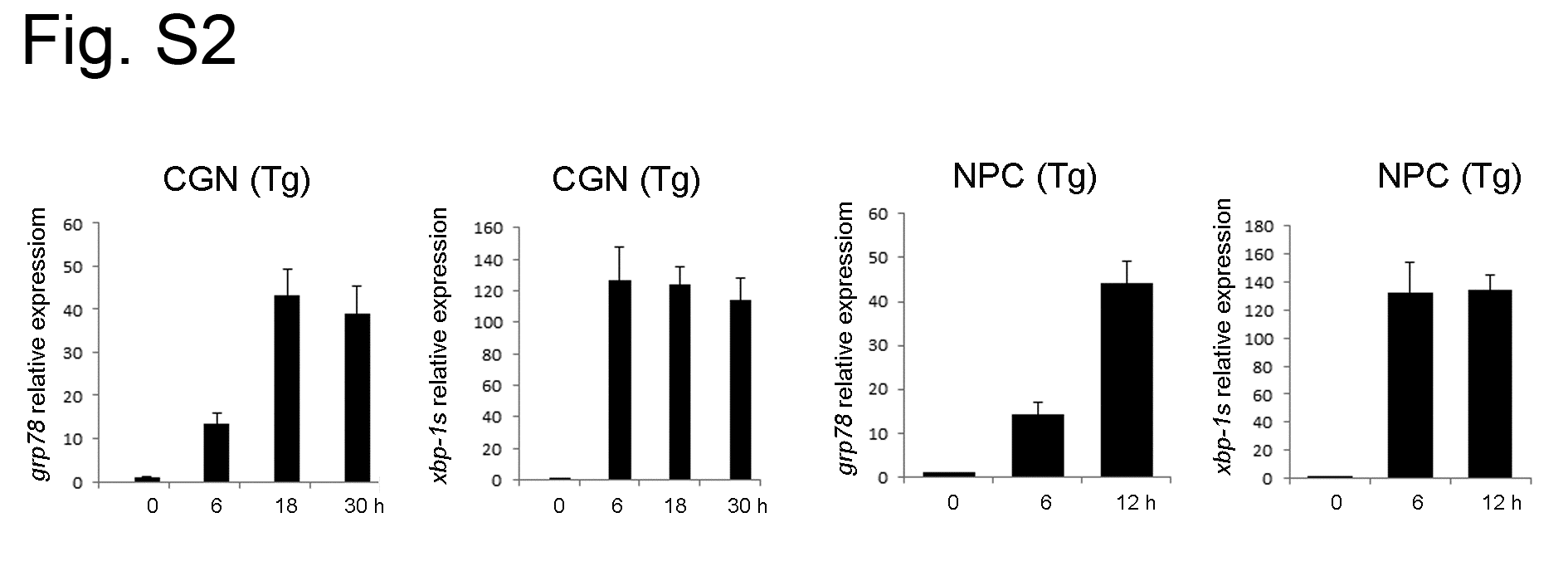

Supplement: Supplementary file 2 — Fig. S2 ER stress induction in mouse primary cells. [file acel0013-0197-sd2.tif]

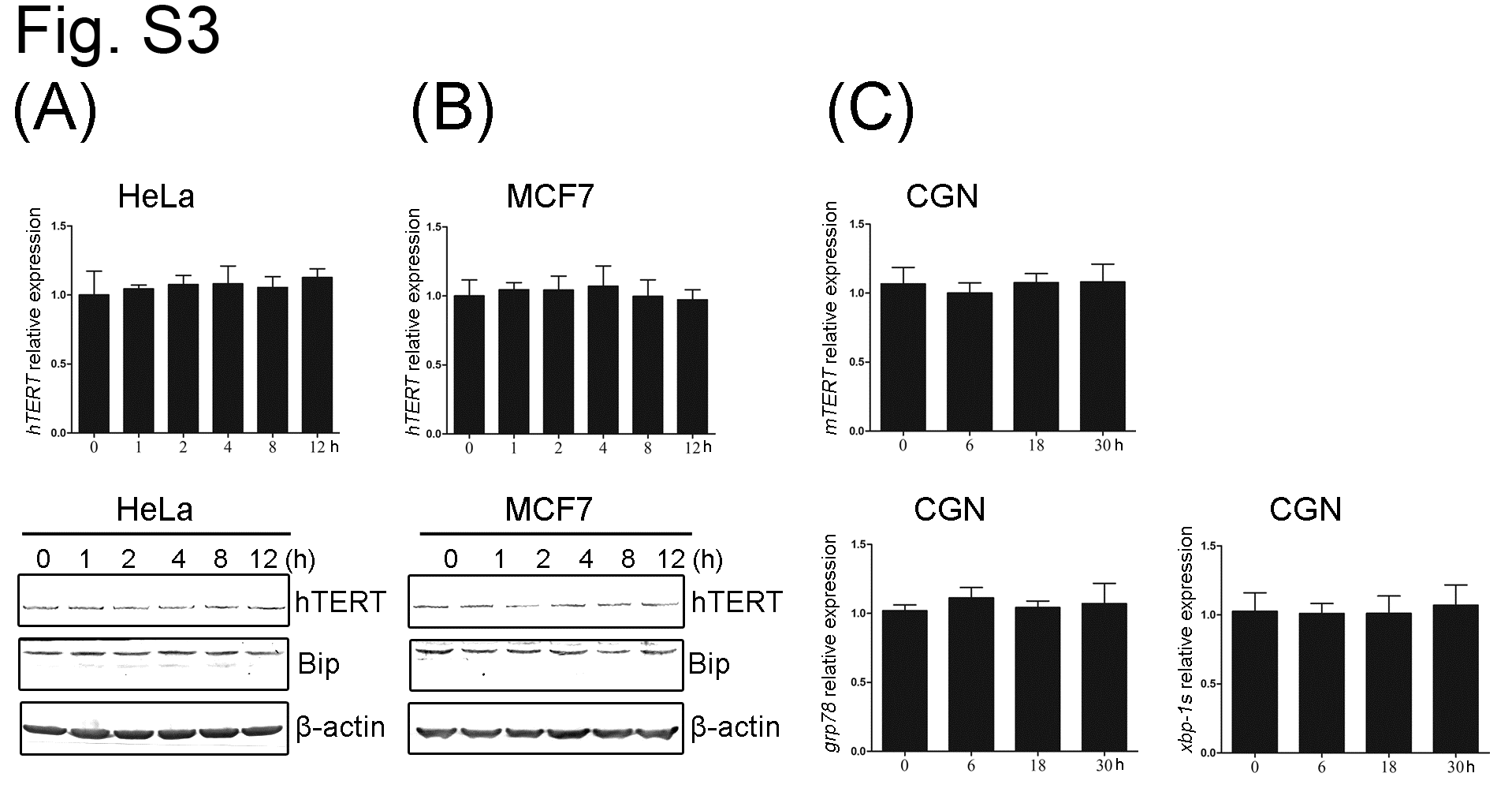

Supplement: Supplementary file 3 — Fig. S3 Effects of the control treatment with DMSO on ER stress induction and TERT up-regulation. [file acel0013-0197-sd3.tif]

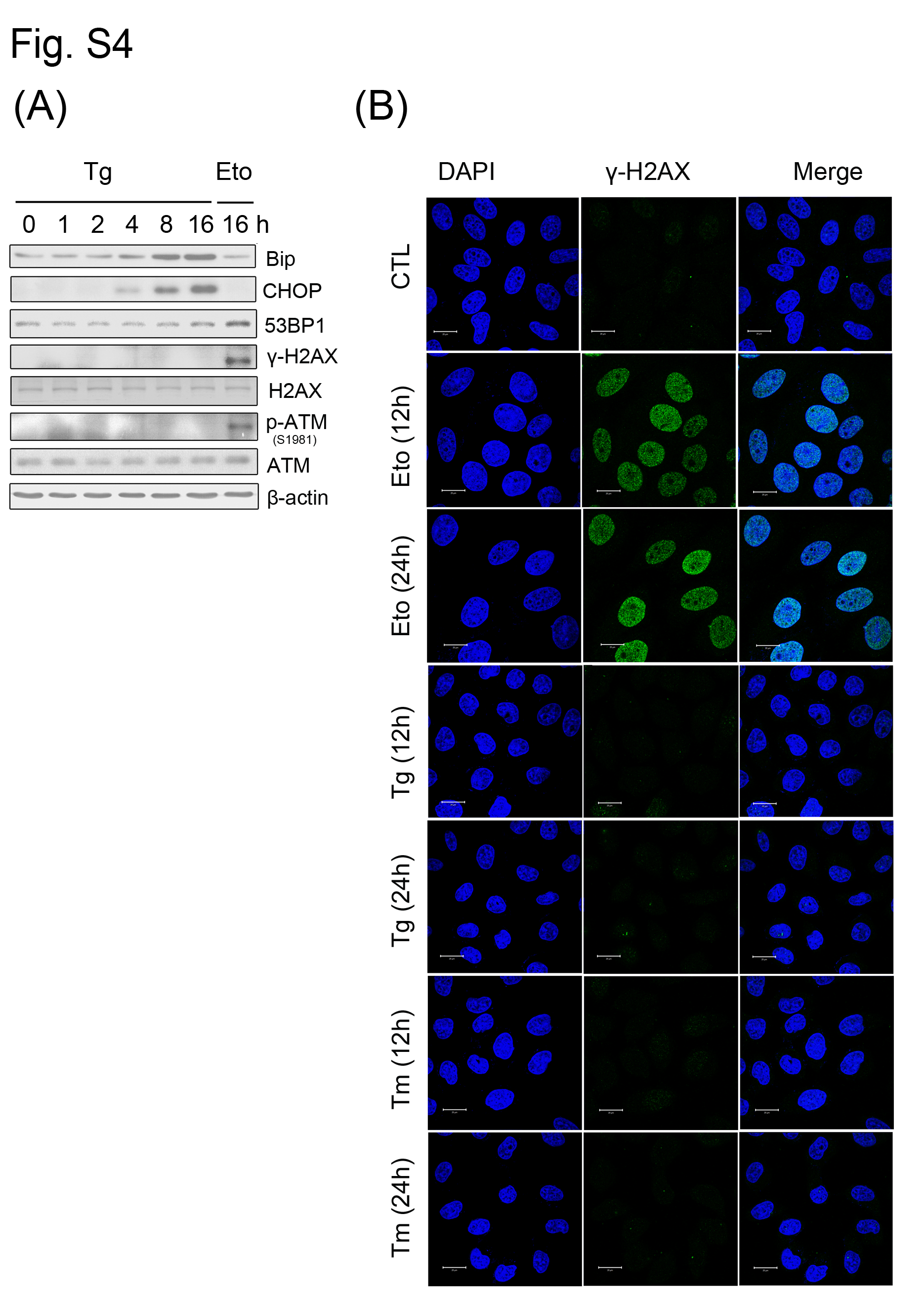

Supplement: Supplementary file 4 — Fig. S4 Absence of DNA damage response in cells under ER stress. [file acel0013-0197-sd4.tif]

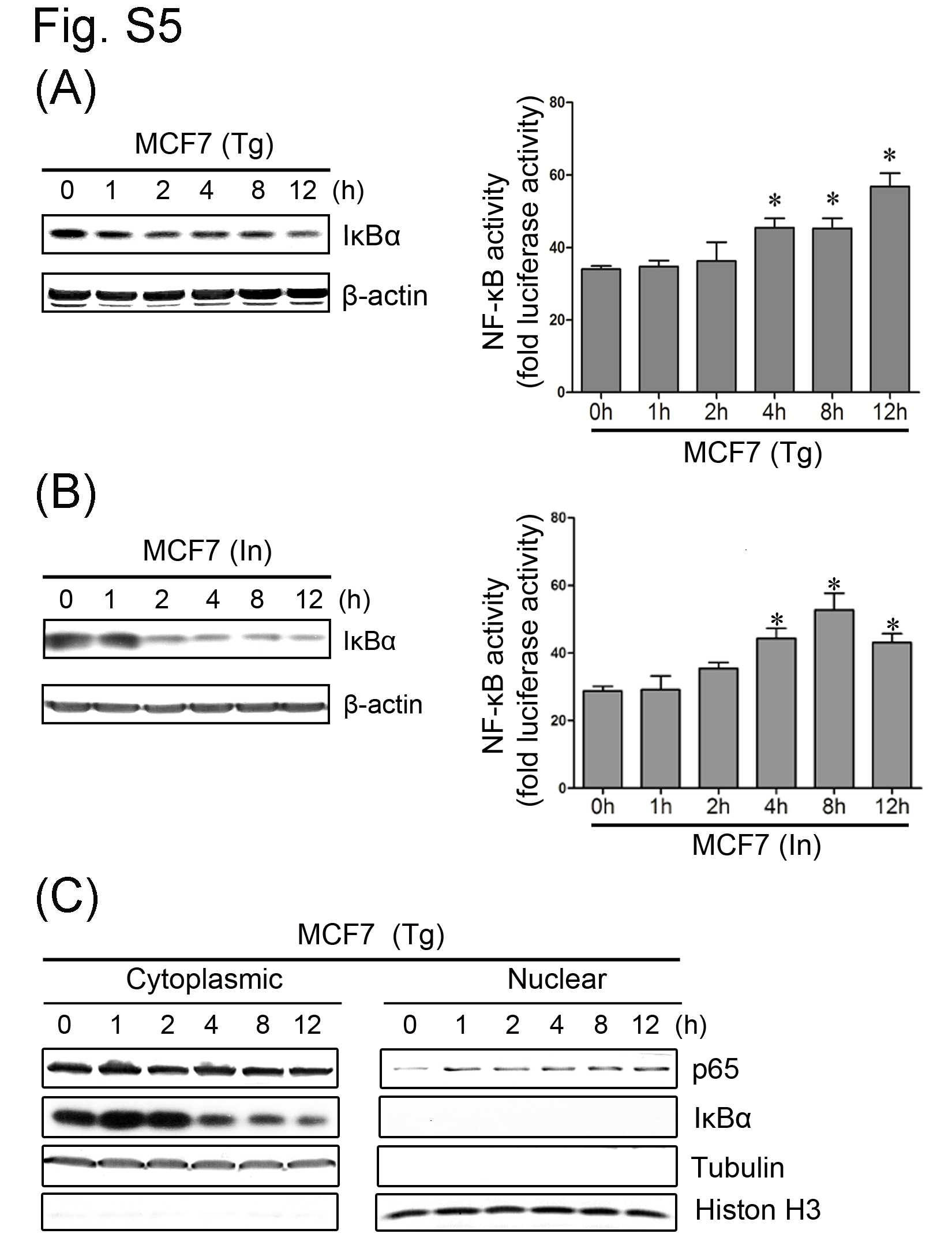

Supplement: Supplementary file 5 — Fig. S5 ER stress enhances the NF-κB activity. [file acel0013-0197-sd5.tif]

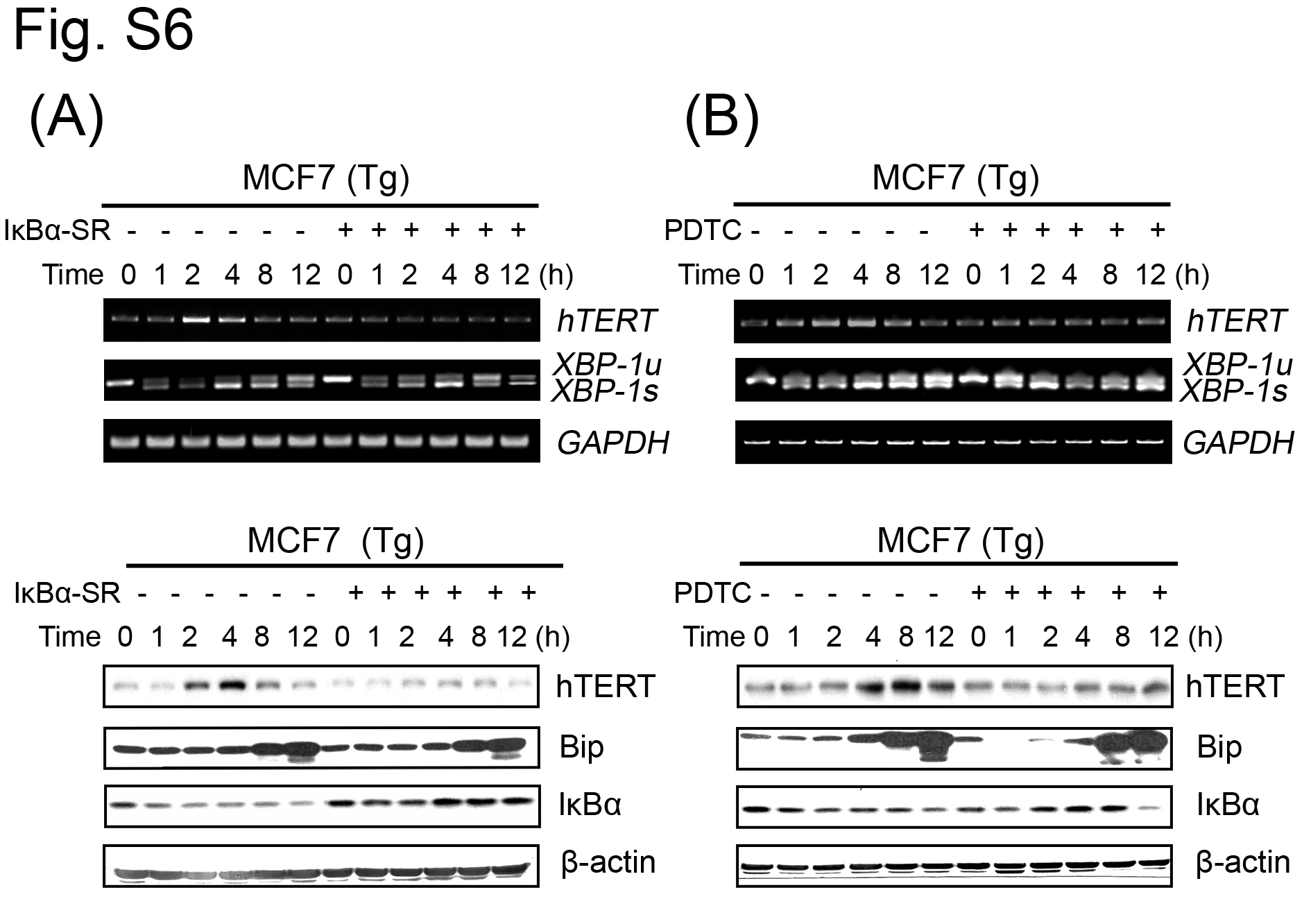

Supplement: Supplementary file 6 — Fig. S6 The NF-κB is required for increased hTERT expression under ER stress. [file acel0013-0197-sd6.tif]

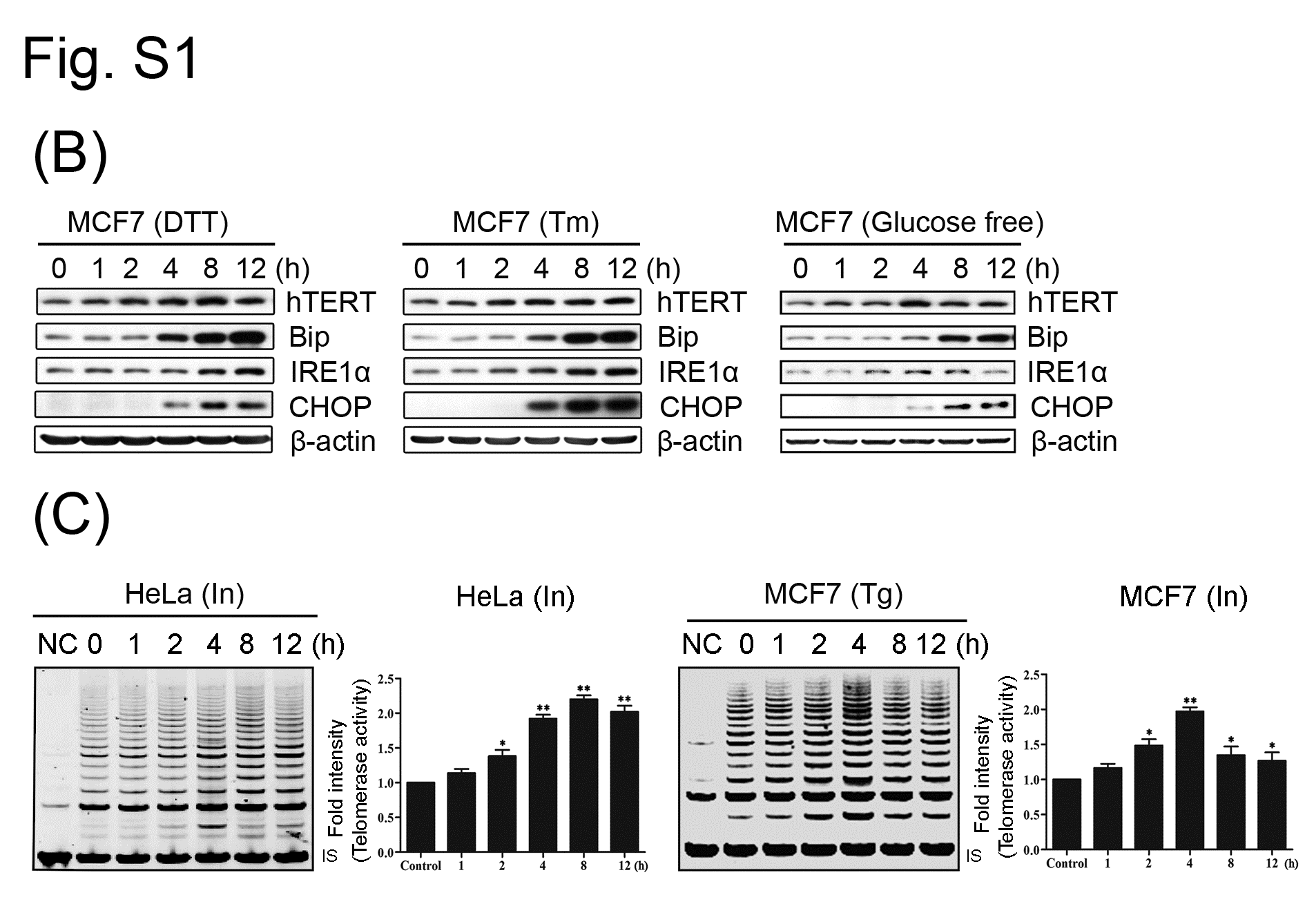

Supplement: Supplementary file 9 [file acel0013-0197-sd9.tif]
