# Supplementary material for: Endoplasmic reticulum stress activates telomerase
Source: Aging Cell. 2013 Oct 22;13(1):197–200. doi: 10.1111/acel.12161 (PMC4326870; doi:10.1111/acel.12161)
Supplement: Supplementary file 7 — Data S1 Experimental procedures. [file acel0013-0197-sd7.docx]

**Experimental Procedures**

*Cell culture, transfection, lentiviral transduction, antibodies and reagents*-MCF7, HeLa and U2OS cell lines were cultured in Dulbecco’s modified Eagle’s medium (DMEM) with 10% fetal bovine serum, 1% L-glutamine, 100 U/ml penicillin and 100 µg/ml streptomycin at 37°C and 5% CO2. Primary neuronal cultures of Cerebellar Granule Neuron (CGN) were established from the postnatal day 7 (P7) WT mouse pups as described (White et al., 1998). CGN cells were cultured in BME supplemented with 10% FCS, 35 mM glucose, and 30 mM KCl for 7 days, followed by treatment with 1 μM Tg for time indicated. Primary cultures of Neural Precursor Cells (NPC) were established as described (Enomoto M et al 2003). Briefly, the brain hemisphere’s ganglionic eminences of embryonic day 14.5 (E14.5) mouse embryos were dissected in cooled HBSS and digested by 5% TE for 3 minutes, transferred the entire suspension to another tube, centrifuged for 5 minutes, 700 rpm and resuspended in DMEM/F12 supplemented with B27 supplement and epidermal growth factor (EGF, 20 ng/mL, Gibco). The cells were adjusted to 1×105 cells/mL in culture flasks and cultured for 5-7 days, followed by treatment with 1 μM Tg for time indicated. The transfection was performed with Lipofectamine 2000 (Invitrogen, Carlsbad, CA, USA) according to the manufacturer’s instructions. hTERT Lentiviral particles were produced in HEK293T cells transfected by Lenti-hTERT and Lenti-K626A viral expression constructs.

Antibodies against the following proteins were used for immunoblotting: β-actin, IRE1α, Bip, IΚBα, 53BP1, ATM, p-ATM (S1981), H2AX, γ-H2AX and CHOP were from Cell Signaling; hTERT was from Abcam (Product ID: Ab32020, Cambridge, UK). AnnexinⅤApoptosis Detection Kit FITC was purchased from BD Bioscience BD (Franklin Lakes, NJ, USA). DL-Dithiothreitol, Thapsigargin, Tunicamycin and Ionomycin were purchased from Sigma (St. Louis, MO, USA).

*hTERT lentiviral constructs and siRNAs***-**The hTERT lentiviral expression constructs Lenti-hTERT and Lenti-K626A were obtained by cloning the full-length hTERT cDNA and an catalytically inactive mutant hTERT K626A into the lentiviral vector pHR-GFP. hTERT siRNAs were purchased from Thermo Scientific (L-003547-00-0020, ON-TARGETplus SMARTpool). The sequences of the siRNAs against hTERT were as follows: 5’-GAACGGGCCUGGAACCAUA-3’, 5’-CGCCUGAGCUGUACUUUGU-3’, 5’-GGUAUGCCGUGGUCCAGAA-3’ and 5’-GCGACGACGUGCUGGUUCA-3’. Control siRNA was purchased from GenePharma (China). The sequences are: sense 5'-UUCUCCGAACGUGUCACGUTT-3', anti-sence 5'-ACGUGACACGUUCGGAGAATT-3'.

*RT-PCR, Quantitative real-time PCR* **-**Total RNA was extracted using TRIzol reagent (Invitrogen) according to the manufacturer’s instructions and followed by cDNAs preparation using M-MLV Reverse Transcriptase (Promega). Real-time PCR reactions were performed in duplicate in SYBR Green Supermix (Bio-Rad, Hercules, CA, USA). The sequences of the primers used for RT-PCR or real-time PCR were as follows:

*GAPDH* 5’-CGGAGTCAACGGATTTGGTCGTAT-3’ and 5’-TGCTAAGCAGTTGGTGGTGCAGGA-3’;

*Xbp-1* 5’-GGAGTTAAGACAGCGCTTGG-3’ and 5’-ACTGGGTCCAAGTTGTCCAG-3’

*hTERT* 5’-TGACACCTCACCTCACCCAC-3’ and 5’-CACTGTCTTCCGCAAGTTCAC-3’.

*mTERT* 5’-CTGAGTCTCACCAGTACAAGTGT-3’ and 5’- TTGGCACCCATGATTTGCCT-3’.

m*Grp78* 5’-TGCAGCAGGACATCAAGTTC-3’ and 5’-CTGCATGGGTGACCTTCTTT-3’.

*mXbp-1s* 5’-GAGTCCGCAGCAGGTG-3’ and 5’-AAGGGAGGCTGGTAAGGAA-3’.

*mGAPDH* 5’- GGTGAAGGTCGGTGTGAACGG-3’ and 5’- TGGTGCAGGATGCATTGCTG-3’.

*Western blot*-Cell lysates were prepared in RIPA buffer. Total protein concentration was measured with BCA kit, an equal amount of samples (20-60 μg) was separated on 12% SDS-polyacrylamide gel and then transferred to PVDF membrane (Millipore). The membranes were incubated at 4°C overnight with primary antibodies.

*Telomerase Assay***-** Cell extracts were prepared and telomerase activity was detected by using TRAPEZE^®^Telomerase Detection Kit (Roche) according to the manufacturer’s instructions, or by using the PCR-based telomere repeat amplification protocol (TRAP).

*Flow cytometry analysis***-**Cells were treated with 2 μM thapsigargin for indicated time, followed by staining with 2-(4-Amidinophenyl)-6-indolecarbamidine dihydrochloride (DAPI) and AnnexinⅤ-fluorescein isothiocyanatc. A total of 10,000 events were acquired by a LSRFortessa flow cytometer (BD Biosciences) and the data were analyzed by Flow Jow software (Tree Star, Ashland, OR).

*LDH activity assays***-**Medium lactate dehydrogenase (LDH) activity was measured with LDH Cytotoxicity Assay Kit (Beyotime, China) according to the manufacturer’s instructions.

*Immunofluorescence-*MCF7 cells were plated on 6-well plate with glass coverslips and treated with etoposide (20 μM) or thapsigargin (2 μM). At indicated time points, cells were fixed with 4% paraformaldehyde in PBS for 15 min and permeabilized in 0.5% TritonX-100. After blocking of non-specific binding sites using 5% BSA, hybridization with γ-H2AX antibody (1:200 dilution) was performed at 37°C for overnight, then with FITC-conjugated secondary antibody at 37°C for 25 min, followed by DNA staining with DAPI (1 μg/ml). Photomicrographs were obtained using a Zeiss LSM710 Confocal Spectral Microscope (Carl Zeiss MicroImaging) and the images were analyzed by using LSM 710 ZEN software.

*Statistical analysis***-**Samples for TRAP assays, apoptosis assays and LDH assays were run in triplicate and experiments were repeated at least three times. Data were analyzed by the Student’s *t*-test for the determination of statistical significance. p<0.05 was considered significant. * represents as p-value <0.05, ** as p-value <0.01.
