# Supplementary material for: Endoplasmic reticulum stress activates telomerase
Source: Aging Cell. 2013 Oct 22;13(1):197–200. doi: 10.1111/acel.12161 (PMC4326870; doi:10.1111/acel.12161)
Supplement: Supplementary file 8 [file acel0013-0197-sd8.docx]

**Supplemental Figure Legends**

**Fig. S1. ER stress up-regulates the expression of hTERT in cancer cells.** (A) MCF7 and HeLa cells were treated with 10μM Ionomycin (In) or 2μM Thapsigargin (Tg). At the indicated time points, cells were harvested for Western blot analysis of hTERT, Bip, IREα and CHOP protein expression. β-actin was used as internal control. The experiment was repeated three times (Experiment 1, Experiment 2 and Experiment 3) and a representative result is shown in upper panel of Fig. 1A. Level of hTERT expression quantified densitometrically from these three independent experiments is shown in lower panel of Fig. 1A. (B) MCF7 cells were treated with glucose free DMEM, 1 mM DTT or 10 μg/mL tunicamycin (Tm) for time indicated. Cell lysates were prepared for Western blot analysis of hTERT, Bip, IRE1α, CHOP protein expression. β-actin was used for internal control. (C) ER stress increased telomerase activity. HeLa and MCF7 cells were treated with 10 μM In and 2 μM Tg respectively. At the indicated time points, cell lysates were prepared for assays of the telomerase activity *in vitro* by TRAP assay.

**Fig. S2. ER stress induction in mouse primary cells.** Primary CGN and NPC cells were treated with Tg (1 μM) for indicated times, respectively. RNA was extracted for analysis of the mRNA expression of *mXBP-1s* and *mGPR78*.

**Fig. S3. Effects of the control treatment with DMSO on ER stress induction and TERT up-regulation.** (A, B) HeLa and MCF7 cells were treated with DMSO, vehicle for ER stress inducers (Tg and In), for indicated times, cells were harvested for real-time PCR analysis of hTERT mRNA expression, and for Western blot analysis of hTERT and Bip protein expression. β-actin was used as internal control. (C) Mouse primary CGN cells were treated with DMSO for indicated times, cells were harvested for real-time PCR analysis of *mTERT*, *mXBP-1s* and *mGPR78* expression.

**Fig. S4. Absence of DNA damage response in cells under ER stress.** (A) MCF7 cells were treated with 2 μM Tg and 20 μM etoposide (Eto) for the indicated time. Cell lysates were prepared for Western blot analysis of Bip, CHOP, 53BP1, H2AX, γ-H2AX, ATM, p-ATM (S1981) protein expression. β-actin was used for internal control. (B) Detection of γ-H2AX foci in cells under ER stress and Eto induced DNA damage. MCF7 cells were treated with 2 μM Tg and 20 μM Eto for the indicated times. Cells were fixed and stained with antibodies against γ-H2AX (green) and DAPI (blue).

**Fig. S5. ER stress enhances the NF-κB activity**. (A, B) The effect of ER stress on the IκBα expression in MCF7 cells. Cells were incubated with 2 μM Tg (A) or 10 μM In (B) in 10% FBS-supplemented DMEM for the indicated time. The levels of IκBα expression were analyzed by western blotting. (C, D) Analysis of NF-κB activity in MCF7 cells under ER stress. MCF7 cells were transfected with the luciferase reporter pNF-κB, purchased from Beyotime Institute of Biotechnology (China). 48 h after transfection, cells were treated with either 2 μM Tg (C) or 10 μM In (D) for the indicated time. The firefly luciferase reporter activity was assayed using the Dual Luciferase Assay System (Promega, Madison, WI) according to the manufacturer’s instructions. Results were expressed as the mean firefly luciferase activity ±s.d. normalized by the co-transfected renilla luciferase reporter activity from three independent experiments. (E) Increased nuclear translocation of the NF-κB p65 under Tg-induced ER stress. Cells were treated with 2 μM Tg for the indicated time. Levels of the NF-κB p65 and IκBα in the cytoplasmic and nuclear fractions were analyzed by Western blotting with antibodies against NF-κB p65 and IκBα. Histone H3 and α-tubulin were used as markers for nuclear and cytoplasmic proteins, respectively.

**Fig. S6. The NF-κB is required for increased hTERT expression under ER stress.** (A) IκBα-SR inhibits the up-regulation of hTERT expression in MCF7 cells under Tg-induced ER stress. MCF7 cells were transfected by the IκBα-SR expression construct or empty vector as control. 48 h after transfection, cell were treated with 2 μM Tg for the indicated time. The expression of *hTERT*, *XBP-1u* and *XBP-1s* mRNAs was analyzed by RT-PCR with specific primers. Levels of hTERT, Bip, IκBα proteins were analyzed by Western blotting with hTERT, Bip and IκBα antibodies. GAPDH and β-actin was used as internal control. (B) PDTC blocks the up-regulation of hTERT expression in MCF7 cells under Tg-induced ER stress. Cultures of MCF7 cells were treated with 2 μM Tg for the indicated time in the presence of PDTC. The expression of *hTERT*, *XBP-1u*, *XBP-1s* mRNAs, and hTERT and IκBα proteins were analyzed as described in (A).
